# Bridging the Access Gap: The Telepractice Experience of Speech Therapists and Audiologists at a Public Health Care Facility in South Africa

**DOI:** 10.5195/ijt.2022.6517

**Published:** 2022-12-13

**Authors:** Shelissa Govender, Annika L. Vallabhjee, Chenay R. Charles, Darike Roesch, Sadna Balton

**Affiliations:** 1 Department of Speech Therapy and Audiology, Chris Hani Baragwanath Academic Hospital, Johannesburg, South Africa; 2 Centre for Augmentative and Alternative Communication, University of Pretoria, Pretoria, South Africa

**Keywords:** Audiology, Public healthcare, Speech therapy, South Africa, Telepractice

## Abstract

South Africa is a low to middle income country (LMIC) with a population of 60 million people. The public health sector serves more than 80% of the population. Chris Hani Baragwanath Academic Hospital is a central level public health care facility situated in Gauteng. The Speech Therapy and Audiology Department provides insight into their telepractice services through a qualitative approach. The onset of the COVID-19 pandemic resulted in therapists exploring telepractice as a sustainable model of service delivery. Therapists and patients encountered many challenges to the implementation of telepractice, however, the commitment of therapists ensured that creative solutions were developed. A comprehensive needs analysis at public health institutions is required to ensure the sustainability of telepractice. A hybrid model (telepractice and in-person consults) holds the potential to reduce the financial burden on patients and increase access to quality patient-centered care.

South Africa (SA) is a low-to-middle-income country (LMIC) with nine provinces and a population of 60 million people (StatsSA, 2021). SA has one public national health system which is state-funded and falls under the National Department of Health (Mathews et al., 2019). There are nine provincial health departments, which serve more than 80% of the population (Pillay, et al., 2020) within a primary healthcare model (Kautzy & Tollman, 2008). South Africa faces a burden of disease that includes HIV, tuberculosis, chronic illness, mental health, injury and violence, and maternal, neonatal, and child mortality (Achoki et al., 2022). There are 3,266 registered speech-language therapists, audiologists, and dually registered speech-language therapists and audiologists of which only 22% are employed in the public health sector (Pillay et al., 2020).

Chris Hani Baragwanath Academic Hospital (CHBAH) is a central level public health facility situated in Soweto, Gauteng in South Africa. It is the largest hospital in Africa serving more than 1.5 million people within the Soweto region while simultaneously serving as a tertiary referral center for much of the Gauteng (11.4 million) and North West (3.7 million) provinces (Parliament of South Africa, 2022). More than 500,000 people in Soweto are reported to be living below the lower poverty line at $48 per person per month (StatSA, 2011).

The Department of Speech Therapy and Audiology (STA) at CHBAH has 14 speech-language therapists, 17 audiologists and 11 dually registered speech-language therapists and audiologists. The STA department provides in-person services across the lifespan for people with communication, hearing, and balance disorders as well as feeding and swallowing difficulties.

The onset of COVID-19 resulted in the immediate termination of outpatient services due to the national lockdown in South Africa which was instituted on March 27, 2020 (Dlamini-Zuma, 2020). This response included the repurposing of staff to the urgent needs required for the management of COVID-19 (Moonasar et al., 2021). Rehabilitation and the needs of people with disabilities were not considered in the public health response to COVID-19 (Banks et al., 2021). Access to in-person speech therapy and audiology services was abruptly halted (Balton et al., 2022). This is apparent in Figure 1 as a sharp decline in outpatient statistics from April 2020 in comparison to 2019.

**Figure 1:**
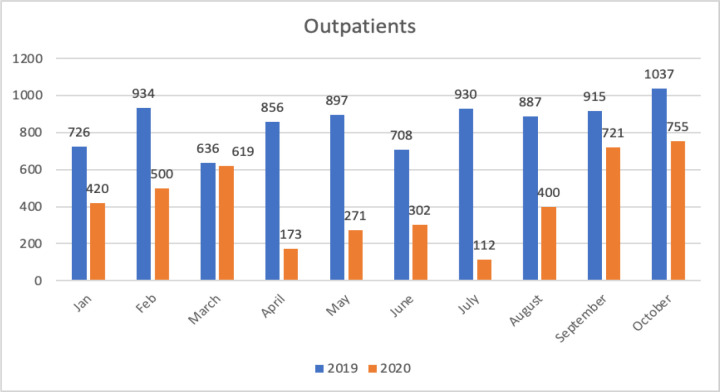
Comparison of Speech Therapy Outpatient Attendances across 2019 and 2020

An alternate model of intervention was explored to ensure continued access to outpatient services. In a brainstorming session with all staff members telepractice was suggested. However, concern was expressed about the feasibility of implementation due to the various constraints faced by our patient demographic. This article sequentially outlines and discusses the implementation of telehealth at STA department at Chris Hani Baragwanath Academic Hospital.

## Planning for Telepractice

Telepractice presented the STA department with an opportunity to increase service platforms to patients in a public health care setting (Balton et al., 2022; Paruk et al., 2022). The statutory body in SA, the Health Professional Council of South Africa (HPCSA) released three amended notices to telepractice guidelines in 2020. These included *Guidelines on Telemedicine in SA*; *Guidance on the Application of Telemedicine Guidelines during the COVID-19 Pandemic*; and *Notice to Amend Telemedicine Guidelines during COVID-19* (HPCSA 1, 2020; HPCSA 2, 2020; HPCSA 3, 2020). Amendments for telepractice included the expansion of telehealth to include telemedicine, telerehabilitation, telepsychology, and telepsychiatry. While a pre-existing therapist-patient relationship was preferable, a new relationship could be started via telepractice (Townsend et al., 2020). These ethical guidelines directed the development of a telepractice protocol to assist with standardizing the service.

Planning also included identifying the training needs of staff in the department. Health care professionals in many countries, including South Africa reported that training for the provision of telepractice was not sufficient at an undergraduate level (Govender & Mars, 2018; Hui et al., 2021). A training program shown in Table 1 was developed to upskill therapists and improve their competence and confidence in providing telepractice.

**Table 1:** Telepractice Training Program

Topics	Description
Orientation on telepractice for community service officers and implementation of telepractice	A description on the different types of telepractice including audiology services. This included a description and examples of synchronous therapy, asynchronous therapy, and remote monitoring.
External training from a digital training expert on how to develop telepractice resources	A training on telepractice and the use of infographics. Guidance on what to include when developing resources to improve digital literacy. This included the skills required to create standardized and professional videos that could be shared with patients. The training also provided therapists with the information required to liaise with external companies.
Training on the Protection of Personal Information Act (POPIA) (*Protection of Personal Information Act*, 2019)	This training focused on ensuring a good understanding of the POPI Act and the implications to be considered within healthcare. This included the legal considerations with record keeping as well as the use of social media.
Ethics regarding online platforms used for telepractice	This focused on compliance and appropriateness of various online platforms (e.g., Whatsapp).
Implementing telehealth - lessons from therapists sharing their experiences with telepractice	A telepractice workshop was hosted to allow therapists from various rehabilitation departments to share their experiences and lessons learned implementing telepractice. Solutions to challenges faced were also shared at this workshop.

In order to initiate telepractice we had to investigate the feasibility of implementation for patients within our context. A questionnaire (Appendix) was developed for this purpose. A random selection of 15 access questionnaires across services in the department were analyzed. Results showed that challenges experienced by patients and families included limited access to data and internet connectivity; this informed the opportunity, frequency, and duration of telepractice. Only 33% (n=5) of patients were aware of free WiFi access points within their respective communities. Sixty-seven percent (n=8) of patients indicated that their data availability was through a “pay as you go” system. The questionnaires revealed that despite the above challenges, 86% (n=13) of patients considered telepractice as a service delivery option. Research looking at the utilization of telepractice within LMIC also found that financial limitations and inadequate technological infrastructure and equipment, as well as digital illiteracy (Chitungo et al., 2021; Mars, 2011; Mars, 2013) affected implementation. The planning phase shed light on the barriers that patients encountered.

Therapists in the STA department also experienced challenges as they did not have access to unlimited internet connectivity as well as a sufficient number of electronic devices to conduct telepractice. A justification for funding was developed and submitted to the hospital management and a mobile communication company. This request was unsuccessful; however, therapists were committed to ensuring access to intervention and therefore independently sourced internet connectivity and cellular devices.

At this stage we were unclear about billing systems for telepractice within a public health setting. Collaboration with various stakeholders, (e.g., hospital management, patient affairs and hospital finances) regarding a billing system for telepractice allowed for improved communication and systems to be established for telepractice. Multiple meetings were held with stakeholders where we shared information on telepractice, including synchronous, asynchronous, and remote monitoring as service options.

Within South Africa's public health system, all patients receiving a social grant, children under six years old and pensioners above the age of 60 years are eligible for free healthcare. All other patients are billed using a specific tariff system within public institutions based on their annual income (SAHRC, 1999; South African Government, 2020). Telepractice provided access to speech therapy and audiology services at a time when patients felt disconnected and marginalized during a period of social isolation (Harkey et al., 2020).

## Clinical Implementation

There is growing support for telepractice internationally (Camden & Silva, 2021, Cleffi et al., 2022; Gefen et al., 2022; Hall & Luechtefeld, 2021) and nationally (Rabe, 2022; Adebayo et al., 2021). In our annual review of services, therapists in the STA department indicated that telepractice allowed for increased caregiver engagement and generalization of activities in the home environment, thereby allowing for improved family centered care (Camden & Silva, 2021, Cleffi et al., 2022; Gefen et al., 2022; Hall & Luechtefeld, 2021). One therapist stated that “through telepractice patients were able to still receive therapy, where this would not have been possible before.”

**Figure 2:**
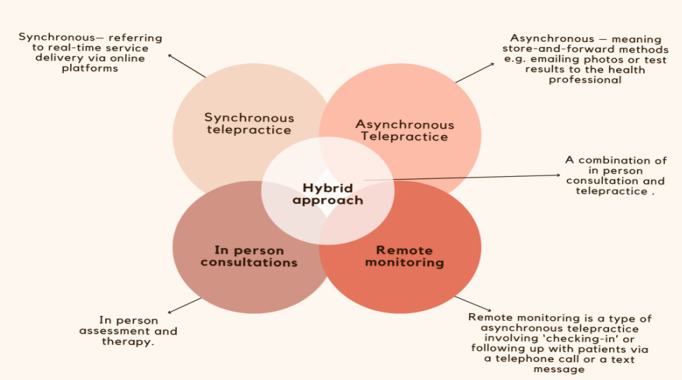
Service Delivery Models used in the STA Department

The clinical implementation of telepractice within the STA department began in April 2020, utilizing three main types of telepractice in conjunction with in-person consultations. The service delivery models used in the STA department are described below, as well as shown in Figure 2.

### Synchronous Telepractice

Synchronous sessions included telephonic consultations and video calls via various platforms (such as WhatsApp video call, Zoom and Microsoft teams). Statistics from April 2020 until June 2022 indicated that 962 synchronous sessions were conducted. These sessions made up 30.97 % of our telehealth practice. Verbal feedback was obtained from patients receiving telepractice to evaluate and improve our services. One caregiver reported that telepractice provided her family with the opportunity to attend weekly synchronous sessions within their home environment. She stated that synchronous sessions provided “flexibility to use objects within our home.” Another caregiver reported she enjoys the sessions as they are helping her child and she is able to make changes on a daily basis. There was agreement among the patients and caregivers that synchronous consultations provided “interactive and flexible” management. Tenforde et al. (2020) found that 93–99% of patients receiving synchronous consultations rated the session positively and consistent with patient-centered outcomes.

The main challenge with accessing synchronous sessions reported by patients and caregivers was that of data costs. Telephonic consultation was therefore the most accessible method identified by patients for synchronous sessions as these posed no additional costs. While this reduced costs for the patients there was limited access to telephones for therapists in therapy rooms. Thus, some synchronous sessions had to be conducted in communal areas. This information was disclosed to patients before a session began and consent was obtained to conduct the session.

There were a number of key lessons learned to conduct successful sessions via a telephone call. Appointment dates and times were set well in advance, to ensure availability of the patient and caregivers or family members as required. Setting therapy aims and goals with patients prior to implementing synchronous sessions was important. It was also important to monitor fatigue levels of patients during these sessions and allow for breaks as needed. Telepractice record keeping was stored as per quality assurance guidelines to ensure that patient confidentiality was maintained (HPCSA, 2021).

### Asynchronous Telepractice

Departmental statistics from April 2020 until June 2022 showed that 902 asynchronous sessions were conducted. Asynchronous therapy sessions were individualized prior to the session with therapists and caregivers jointly identifying specific goals to be targeted. The patient or caregiver made a video recording showing their implementation of strategies within their home environment. Due to data affordability the patient or caregivers sometimes shared these video recordings at a later stage when they had access to data or were within a free WiFi zone. After the therapist and caregiver reviewed the video, both were able to engage and reflect on the implementation of therapeutic strategies and progress. This feedback was done via a telephone call, voice note, or SMS (short message service). This information was also documented in the patient's file. A disclaimer informed patients that the information shared was specific to them or their child and should not be shared with others.

The video resources for patients with hearing impairment included: troubleshooting for hearing aids, cochlear implants, assistive listening devices and aural rehabilitation resources (i.e., listening checks, home programs). Speech-language therapy and dysphagia resources included: videos on language and listening stimulation within the home context, reflux management, troubleshooting for patients with gastrostomies, tracheostomies, and laryngectomies, as well as exercises for patients with speech disorders. Some resources were translated into other languages like isiZulu and Sesotho. A review of our videos by an expert in digital training highlighted the need for standardization in terms of background, lighting, sound, and pacing of information. A departmental guideline was developed with recommendations on how to create videos and resources. This also included information on improving the technical quality of both synchronous and asynchronous sessions.

Data remained a challenge, but patients and caregivers addressed this by sending and/or downloading videos or home programs when they had access to data or were in a free WiFi zone (such as a shopping mall, local library, or school). The main advantage of asynchronous sessions was that patients could engage with therapy material and resources at the times that were most convenient for them. One caregiver stated that “feedback on the videos helped me to identify aspects and development that I as a parent have not seen before.” Another patient reported that “these sessions at home were more relaxed.” While the advantages of asynchronous sessions were identified by therapists and patients some felt that the “personal touch” was missing.

### Remote Monitoring Telepractice

Remote monitoring was started in April 2020 with a total of 1,242 remote monitoring sessions conducted from April 2020 to June 2022.

Remote monitoring refers to the ability to monitor the patient's communication, dysphagia, hearing, and balance through a phone call. This facilitated a space to connect with patients when in-person therapy was halted during the initial lockdown. Remote monitoring reduces patients' travel costs and risk for infection (Cheng et al., 2022; Annie et al., 2020). Therapists indicated that remote monitoring in the early stages of the pandemic provided opportunities for patients to acquire information about COVID-19, rehabilitation, and their general medical management. It also enabled therapists to inquire about a patient's adherence to medication and provide reminders about childhood vaccinations.

Remote monitoring also allowed for early identification of problems. One parent highlighted this when stating: “they advised me when I could come urgently into the repairs clinic, like when my child needed new ear molds for her hearing aids, and I didn't have to wait for months for my regular appointment.”

### Hybrid Approach

A hybrid approach to service delivery is a combination of in-person consultation and telepractice. This approach was offered to patients and included the management options of synchronous, asynchronous, remote monitoring and in-person consultations (Krumm & Syms, 2011; Swanepoel et al., 2010; HPCSA, 2014; Swanepoel & Hall, 2010). A hybrid model of service delivery allowed for increased access and support to patients and their families. Patients appreciated being called after an in-person consultation; as this made them feel supported and empowered.

One of the therapists offering a hybrid therapy approach stated:

“For the families the uncertain times continue but as the lockdown level eases there is more support from family, friends, and the possibility for in-person consultations. The families are so resilient and have been able to adapt to a new service delivery model. Most families enjoy telepractice and the ideal for the families is to continue with tele-rehab with once a month in-person consultation”.

The utilization of a hybrid service delivery model is essential to addressing the challenges experienced in South Africa wherein patients are unable to receive regular therapy due to human resource challenges and financial constraints (Benson, 2020; Coco, 2020; Khoza-Shangase et al., 2021).

### Ensuring the Sustainability of Telepractice

Telepractice reduces travel time, alleviates travel costs, and allows patients that live in geographically remote areas to access health care services (Wales et al., 2017). Telepractice allowed us to continue service provision during the pandemic. An initial solution to ensuring that our patients continue to receive intervention is now a viable future option for our patients. We have discovered that telepractice is possible within the public health sector when the relevant technology, resources, and support are available.

The initiation of a telepractice committee within the STA department facilitated a streamlined approach to resource development, training, record keeping, capturing of statistics, increased telepractice awareness, and improved service provision. These measures will enable our program to continually evaluate telepractice by obtaining therapist and patient feedback, analyzing therapy outcomes, and upholding compliance to relevant telepractice guidelines.

For telepractice to become the norm across public health institutions there needs to be a clear directive on telepractice expectations from the Department of Health that includes guidelines, standardized systems to capture relevant statistics, and continued stakeholder engagement.

To ensure the sustainability of telepractice, undergraduate training needs to provide students with opportunities to develop contextually relevant skills both in theory and practice. This will ensure that therapists entering the workplace are equipped to provide effective telepractice in the South African context.

There must be a commitment from therapists to continue developing their knowledge and skills and contribute to relevant research outcomes on the implementation of telehealth within a LMIC. We have already begun to share our knowledge and skills gained through various platforms and stakeholders.

Understanding the efficacy of the hybrid model of service delivery is essential to delivering quality telepractice services within a STA department. This may be achieved through research and conducting telepractice feedback questionnaires with patients and caregivers.

## Conclusion

Therapists and patients encountered many challenges regarding the implementation of telepractice. However, the commitment of the therapists ensured that creative solutions were developed. Challenges still exist in terms of digital poverty and the lack of an infrastructure that supports the successful implementation of telepractice.

A hybrid service delivery model of telepractice has the potential to be reproduced in other public health facilities. The hybrid model holds potential to reduce the financial burden on patients and increase access to quality patient centered care (Benson, 2020; Coco, 2020; Camden & Silva, 2021, Cleffi et al., 2022; Gefen et al., 2022; Hall & Luechtefeld, 2021; Khoza-Shangase et al., 2021).

Our telepractice service delivery model described herein aligns with the call for “quality health care for all” in the National Development Plan of South Africa (NDP, 2017). Advocating for support from government departments is critical so that adequate budgetary planning and allocation can take place. A comprehensive needs analysis at public health institutions is required, to determine the human resources, equipment, and logistical support necessary to sustain telepractice.

## Figures and Tables

**Figure:**
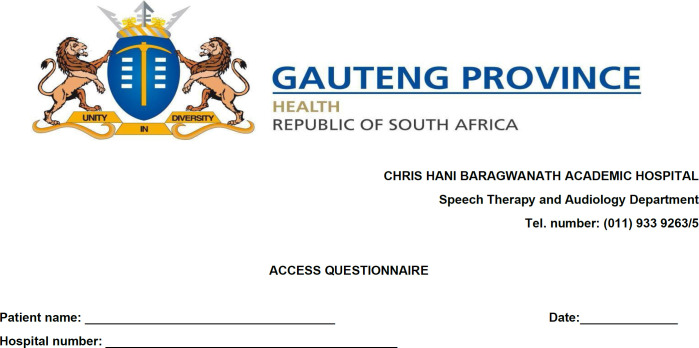
Speech Therapy and Audiology Access Questionnaire

## Appendix

The Speech therapy and Audiology department aims to provide treatment in the most effective manner, whilst maintaining safety for both the patient and therapist. There are various treatment modalities available and you may choose one or a combination of the following options. Please note that the initial in-person consultation session is conducted within the Speech Therapy and Audiology Department, thereafter any of the options listed below can be selected.

**Table:**

**Please select the option/s that you would prefer:**	X
*Telephonic Therapy* Requirements: A landline or cell phone with good reception and battery charge. How it works: Your therapist will arrange an appointment at a date and time that suits you. You will be contacted telephonically and the entire therapy session will be conducted over the phone. Sessions can vary in length from 10–60 minutes, depending on the treatment being provided. You will receive an electronic copy of your therapy session via email or SMS.	
*Live video calls* Requirements: Access to a stable internet connection (data bundles, WiFi, or free WiFi at a local public place), a smartphone or computer. How it works: Your therapist will arrange an appointment at a date and time that suits you. A video call will be set up on either: 1.) Whatsapp, 2.) Zoom, 3.) Skype, 4.) Microsoft Teams, 5.) Google Meets (Please underline preferred platform). Sessions can vary in length from 20–60 minutes, depending on the treatment being provided or your data. You will receive an electronic copy of your therapy session via email.	
*Electronic home programmes* Requirements: Occasional access to the internet, either on a smartphone or computer. A smartphone that can send/receive MMS's or videos (email or Whatsapp). Printing facilities are beneficial but not essential. How it works: Your therapist will send you a home therapy programmes, via email, MMS or Whatsapp. The home programme may be in the form of a text document or a video. You may also be requested to take short videos and send them to your therapist for review and feedback. You will receive telephone contact from your therapist to follow up on the outcome/success of the home programme.	
*In-person sessions:* Requirements: You will need to commit to at least one session per month. If your hospital classification is H1, H2 or H3, you will be required to pay a certain amount for your hospital stamp at the registrations department. How it works: You will receive face-to-face treatment within the Speech Therapy department for approximately 75 mins (+15 mins admin time) during an allocated appointment slot. You will receive home programmes as handouts at the end of your session.	

**Internet access questionnaire:** (Only complete if patient is interested in tele-rehab)

**Table:**

Do you have a landline at home?	If yes, tel: no: ____________
Do you have access to a cell phone?	yes – my own yes – someone in my family no – don't have a cellphone
Do you have access to any of the following electronic devices?	smartphone Laptop tablet/IPad computer
Do you have internet access at home?	yes no
If yes to question above, what type of internet	Pay-as-you-go data bundles Predetermined data on contract Capped WiFi Uncapped WiFi
Are you aware of where you can access free WiFi/internet nearby your home?	yes no
If yes to the question above, where and how far do you have to travel?	
Do you have an email address? email: _____________	Yes - my own Yes - someone in my family No
Is there someone at home who can help you with completing a telerehabilitation programme?	
Is there someone at home who can assist with setup and troubleshooting of devices (such as setting video call, zoom etc.,) required for participating in a telerehab programme?	
Does your electronic device have a front-facing camera for video calling?	
